# Carbon Emissions Trading and Green Technology Innovation—A Quasi-natural Experiment Based on a Carbon Trading Market Pilot

**DOI:** 10.3390/ijerph192416700

**Published:** 2022-12-12

**Authors:** Weiming Liu, Yating Qiu, Lijiang Jia, Hang Zhou

**Affiliations:** 1 School of Shipping Economics and Trade, Guangzhou Maritime University, Guangzhou 510725, China; 2 School of Economics, Jiangxi University of Finance and Economics, Nanchang 330013, China; 3 College of Economics and Management, Harbin Engineering University, Harbin 150001, China

**Keywords:** carbon trading, green technology innovation, differences-in-differences

## Abstract

Carbon emissions trading policy has received widespread attention from scholars as a core policy tool to reduce carbon emissions. While most scholars have previously focused on the carbon emission reduction effect, this paper investigates the impact of carbon emissions trading policy on green technology innovation using a differences-in-differences method based on provincial panel data from 2005–2019, using a carbon emissions trading pilot as a quasi-natural experiment. The findings show that the policy can significantly promote green technology innovation, but with a lagged effect, and this finding still holds in the robustness test. Further heterogeneity analysis reveals that the stronger the human capital, the stronger the intellectual property protection and the stronger the marketization with better policy effects. In addition, carbon emissions trading policy can indirectly affect green technology innovation by influencing research investment.

## 1. Introduction

In recent years, with the increasing frequency of extreme weather such as heat waves, droughts, floods, and typhoons, the threat of global climate change to the sustainable development of ecosystems and human beings has become a common concern of the international community. To meet this serious challenge, the parties to the United Nations Framework Convention on Climate Change have signed the Kyoto Protocol and the Paris Agreement, committing themselves to limiting the global temperature increase to 2 °C. As a responsible major country, China put forward the goal of “peaking greenhouse gases before 2030 and striving to achieve carbon neutrality before 2060” (referred to as the “dual carbon” goal) in 2020 from the perspective of building a community with a shared future for mankind. The proposal of the “double carbon” goal is a solemn commitment to the international community and an inherent requirement for China’s high-quality development. As an important market-based emission reduction tool, carbon emissions trading is one of the core policy tools to achieve the “dual carbon” goal and the low-carbon transformation of China’s economy [1].

China’s carbon emissions trading has taken about ten years to go from local pilot to full-scale establishment. In October 2011, the National Development and Reform Commission issued the Notice on the Launch of Carbon Emissions Trading Pilot, approving the launch of the carbon emissions trading pilot in seven provinces and cities, namely, Beijing, Shanghai, Tianjin, Chongqing, Guangdong, Hubei, and Shenzhen; since June 2013, Shenzhen, Shanghai, Beijing, Guangdong, and Tianjin Hubei and Chongqing have successively implemented carbon emission trading policies one after another. have continued with the policy. At the end of 2021, the national carbon emissions trading market went online and the power generation industry became the first industry to be included in the carbon emissions trading market, covering approximately 4.5 billion tons of CO2 emissions, making it the largest carbon emissions trading market in the world [2], and key industries such as petrochemicals and steel will also be included in the national carbon emissions trading market in the future.

The carbon emissions trading mechanism was originally designed to achieve carbon emission reduction targets at minimal cost, inducing enterprises to undertake operational reforms, energy transformation and R&D investments, accelerating green technological innovation and ultimately achieving the win-win goal of economic growth and environmental protection. In the ten years from 2011 to 2021, China’s carbon emissions trading pilot covered more than 20 industries and nearly 3000 enterprises, with a cumulative turnover of more than 10 billion yuan [3]. The carbon emissions intensity in the pilot areas fell significantly, and the growth rate of total carbon emissions slowed significantly, with China’s carbon emissions intensity in 2020 falling by 48.4% compared to that in 2005, exceeding the climate action targets to which China committed [4]. However, did the achievement of the previous targets come from corporate production cuts or green technology innovation? Is the role of pilot carbon trading important in this? What is the mechanism of their role? Is there heterogeneity in the policy effects of carbon trading pilots in different regions? Do carbon trading pilots have an impact on neighboring cities? There is little discussion of this in the existing literature, and these are all questions that need to be answered.

The paper is organized as follows: Section 1 is the introduction; Section 2 is the literature review; Section 3 includes the theory and hypotheses; Section 4 describes the data sources and model design; Section 5 is the empirical results; Section 6 presents the discussion. Section 7 concludes.

## 2. Literature Review

The innovation compensation effect of environmental regulation suggests that reasonably designed environmental policies can provide incentives for firms to innovate and use green technologies, and that the resulting increase in productivity can at least partially compensate for the compliance costs of environmental regulation. This theory is known as the “Porter hypothesis” [5], as it was first proposed by Harvard professor Porter. “The Porter Hypothesis” has been the subject of much debate since its inception, with a focus on what constitutes a well-designed environmental policy. Although environmental policy tools are diverse and their effectiveness varies, after years of practice, it has been found that market-incentivized environmental policies are generally less costly, more inducing of green innovation and less socially shocking than command-and-control environmental policies [6].

The root of the climate problem is externalities, and the core mechanism of market-incentivized environmental policy is to internalize environmental costs through the imposition of environmental taxes or emissions trading [7]. According to Coase’s theorem, where transaction costs are zero or small, externalities can be addressed through market mechanisms as long as property rights are clear. Carbon emissions trading usually involves the government estimating the maximum amount of carbon emissions that can be emitted from a certain region’s environmental capacity and dividing it into a number of emission shares, which are given to enterprises on a paid or unpaid basis so that they can freely trade carbon emission rights in the secondary market according to their emissions [8]. The direct effect of carbon trading is twofold: firstly, the cost-push effect, in which enterprises will increase their compliance costs if their emissions exceed their allowances after being bound by the carbon trading mechanism; secondly, the revenue-incentive effect, in which enterprises can earn revenue by selling their excess carbon credits [9]. The implementation of carbon emissions trading policies significantly reduces regional carbon emissions intensity and has a significant emission reduction effect [10]; its realization path mainly lies in promoting industrial structure upgrading and improving resource allocation efficiency, in which technological innovation is manifested as a masking effect [11]. In terms of practical effects, the US carbon emissions trading market and the EU carbon emissions trading system (EU ETS), for example, have received good results. For example, the EU ETS has significantly promoted renewable energy technology innovation, as the system uses part of the auction revenue from carbon market allowances for renewable energy R&D and project investment, reducing the risk exposure of renewable energy enterprises and establishing a relatively good investment environment, and has helped the development of renewable energy firms that are not yet fully cost-competitive and initially dependent on government financial subsidies, allowing member countries to transform their energy systems towards cleaner, renewable energy sources [12]. A similar effect is seen in China, where Song Deyong et al. found that carbon trading significantly promotes corporate green innovation by matching data on key emission control enterprises in the carbon trading pilot with patent data on listed companies [13].

In summary, the existing research on the policy effect of carbon emissions trading has formed a relatively systematic system, which has certain reference value for this paper; however, its policy is mainly reflected in the research on its emission reduction effect [14], and there is relatively little research on its promotion of green technology innovation. The existing research on the impact of China’s carbon emissions trading pilot on technological innovation is mainly concentrated at the micro-enterprise level, and is relatively rare at the macro level, and the research on the spillover effect of pilot policy space is more scarcer. Based on this, the possible contributions of this paper are as follows: First, based on the data of China’s provincial green patents from 2005 to 2019, the pilot cities of carbon emissions trading are used as quasi-natural experiments to evaluate the green technology innovation effect of carbon emissions trading policies, and further analyze whether there are spatial spillover effects, thereby enriching the relevant literature of the “Porter hypothesis”. Second, the mediation effect model was constructed, the impact mechanism of carbon emissions trading policy on regional green technology innovation was discussed, and the policy effect of market-incentivized environmental regulation tools was analyzed through heterogeneity analysis.

## 3. Theory and Hypotheses

Neoclassical economic theory suggests that environmental regulations increase the cost of production and the cost of dealing with pollutants, thus reducing the productivity of enterprises; therefore, enterprises are constrained by the environment and limit their normal production activities, including green innovation activities. For example, Shen Hongtao found through empirical tests that the implementation of carbon emissions trading policies can effectively lead to carbon emission reductions, but firms mainly reduce carbon emissions through short-term actions such as reducing production, rather than long-term emission reductions by investing in emission reduction technologies to achieve cleaner production [15]. Porter’s hypothesis suggests that environmental regulations do not necessarily increase the production burden of firms but instead force them to engage in green technological innovation, which can offset the losses caused by environmental regulations. Carbon emissions trading is a special kind of environmental regulation policy that allocates carbon resources by trading carbon allowances in the carbon market. When an enterprise’s carbon emissions exceed a given carbon quota, it needs to buy carbon emission rights in the market to make the enterprise comply with the policy. This can be used for green technology research and development, thus promoting green technology innovation in pilot regions with carbon emissions [16]. Therefore, this paper proposes the following hypotheses:

**Hypothesis 1 (H1).**  *Carbon emissions trading system can facilitate innovation in green technologies for carbon pilots*.

**Hypothesis 2 (H2).**  *Carbon emissions trading system can promote green technology innovation in carbon pilots by increasing investment in green technology research and development*.

Based on the national level, as the level of human capital, the level of market development, and the intensity of intellectual property protection vary from one carbon trading pilot to another, the effect of the carbon trading policy pilot also has some regional differences. For example, Shanghai, Beijing, Guangdong, and Hubei are the provinces with more universities and have relatively more human capital; thus, the pilot carbon emissions trading policy will have a relatively good effect on promoting green technology innovation [17]; from the level of market development, the higher the degree of marketization, the easier it is to sell the carbon emission rights saved through innovation, and the more incentives enterprises will have to invest, which creates good conditions for green technology innovation [18]. In terms of the level of intellectual property protection, the level of protection varies from region to region, and so does the incentive for enterprises to engage in technological innovation [19]. The effect of carbon trading policies on green technology innovation varies from region to region. Therefore, the effect of the system is heterogeneous due to differences in the level of human capital, the level of market development, and the intensity of intellectual property protection. Based on this analysis, this paper proposes the following:

**Hypothesis 3 (H3).**  *Differences in the level of human capital, the level of marketization, and the intensity of intellectual property protection lead to differences in the effectiveness of carbon emissions trading policies in promoting green technology innovation in different regions*.

Because of the interconnectedness between regions, the impact of one region may have a corresponding impact on neighboring provinces. This paper argues that when a region first implements the pilot of carbon emissions trading, the corporate agents of other provinces will perceive the wind direction of the environmental regulation and instigate long-term planning, because the pilot area promotes green technology research and development investment through carbon emissions trading, and then promotes the green innovation of enterprises; then, companies in other provinces will imitate the corporate strategy made by enterprises in the pilot area, thereby increasing the green technology innovation of neighboring provinces, creating a positive radiation effect.

**Hypothesis 4 (H4).**  *Green technology innovation in pilot areas will have a positive radiation effect on green technology innovation in neighboring provinces*.

## 4. Data Sources and Model Design

### 4.1. Selection of Variables and Data Sources

This paper uses provincial panel data from 2005–2019 data as the initial sample to evaluate the effect of the pilot policy of carbon emissions trading system; the data come from the China Statistical Yearbook, China Industrial Statistical Yearbook, China Science and Technology Yearbook, etc. Since Shenzhen belongs to Guangdong Province, Shenzhen is included in the data of Guangdong Province in this paper, where the total import and export is manually converted from USD to RMB.

Dependent variable: green technology innovation Invent, which is measured in this paper by the number of green invention patent applications /1000, referring to the method of Wu gezhi [20].

Core independent variable: carbon trading pilot regions interaction term $DID_{ct}$($treatment_{c}\;$* $post_{t}$). The seven official carbon trading pilots in China only started trading in the second half of 2013 and the first half of 2014. The binding impact of the carbon trading market on emitters depends on its compliance mechanism and the actual penalties for non-compliant enterprises; on the other hand, it takes time for policies to go from implementation to policy effects and for green innovations to emerge. Therefore, the pilot can only play a substantial role after the official opening of the market, and there is a certain time lag—according to the results of the parallel trend test, the policy effect lags two years; so, the policy node is set in 2015. The $treatment_{c}$ is the policy group dummy variable and $post_{t}$ is the time dummy variable. Within the carbon emissions trading pilot, $treatment_{c}$ = 1; outside of the pilot, it is 0; when the time is later than 2015, *post_t_* = l; otherwise, it should be 0.

Mediating and moderating variables: In this paper, research input (*Input*) is selected as the mediating variable, and R&D expenditure of industrial enterprises above the scale is selected as the measure by referring to Zhang Jiangxue et al. [21]. In this paper, the most common linear interpolation method is used to fill in the missing values due to the existence of R&D expenditure of industrial enterprises above the scale.

In this paper, the level of human capital (*HC*), the degree of marketization (*Market*), and the level of intellectual property protection (IPR) are selected as moderating variables, where HC indicates the level of human capital in a city, measured by the percentage of the city’s employed population with education level above the specialist level; the degree of marketization (*Market*) is measured by Fan Gang’s Marketization Index report [22]. Trans indicates the intensity of a city’s level of intellectual property protection, as measured by Hu Kai [23], using the value of technology market transactions in the city/GDP of the region.

Control variables: In this paper, 5 control variables are selected. Referring to the studies of other scholars in related fields [24,25,26,27], the degree of openness to the outside world (*Open*), enterprise size (*Scale*), level of economic development (*GDP*), investment in science and technology (*Tec*), and industrial structure (*STR*) were selected as control variables in this paper. The degree of openness to the outside world is measured by dividing total imports and exports by GDP, and trade activities have a technology diffusion effect, which may have an impact on green technology innovation of enterprises. The more developed the economic level, and the better the business environment and the institution is, the more conducive it is for enterprises to obtain funds for green technology innovation, which can create a favorable environment for enterprises to innovate. The input of personal scientific and technological (*Tec*) is expressed as the full-time equivalent of R&D personnel (person-years) in industrial enterprises above a certain size. The industrial structure (*STR*) is a reflection of the level of regional economy and industrial development, measured by the proportion of the added value of tertiary industry to regional GDP.

### 4.2. Descriptive Statistics

The specific variables involved in this paper and their descriptive statistics are presented in Table 1.

From the above descriptive statistics, it can be seen that the minimum value of green invention patents is 0 and the maximum value is 31,391 between 2005 and 2019, which is a large increase; in addition, the investment in research has also increased a lot, which justifies the data and the hypothesis of this paper.

### 4.3. Model Design

This paper uses differences-in-differences to estimate the impact of the establishment of the carbon emissions trading pilot on regional green technology innovation, which was established in 2013 as a natural experiment. Controlling other factors, the differences-in-differences was used to test whether there was a significant difference in regional green technology innovation between the treatment and control groups before and after the establishment of the carbon emissions trading pilot. The model was set up as follows.

(1) $$Invent_{ct}=\beta_{0}+\beta_{1}DID_{ct}+\beta_{2}Control_{ct}+\eta_{c}+\gamma_{t}+\varepsilon_{ct}$$

In Equation (1), $Invent_{ct}$ is the explanatory variable, containing the number of green invention patent applications/1000, which is used to measure the level of green technology innovation. $DID_{ct}$ is the main explanatory variable, $DID_{ct}=treatment_{c}*post_{t}$, in the sample period; if c is a carbon emissions trading pilot, then $treatment_{c}$ = l; when the year is greater than 2015, $post_{t}$ = l. The treatment group in this paper is the carbon trading pilot regions.

Pilot region and the control group is the non-carbon trading pilot region. The subscripts c and t represent province and time, respectively. $Control_{ct}$ denotes the control variables that vary with province and time, $\eta_{c}$ denotes province fixed effects, $\gamma_{t}$ denotes time-fixed effects, and $\varepsilon_{ct}$ denotes the error term, where the coefficient is $\beta_{1}$. The magnitude of the coefficient is the impact of the carbon emissions trading system on regional green technology innovation. If $\beta_{1}$ is greater than 0, then the carbon emissions trading system has a positive effect on regional green technology innovation; if not, the opposite is true.

In order to test whether there is heterogeneous variation in this policy, this paper extends the previous model by constructing the following model:

(2) $$Invent_{ct}=\beta_{0}+\beta_{1}DID_{ct}*HC_{ct}+\beta_{2}Control_{ct}+\eta_{c}+\gamma_{t}+\varepsilon_{ct}$$

(3) $$Invent_{ct}=\beta_{0}+\beta_{1}DID_{ct}*IPR_{ct}+\beta_{2}Control_{ct}+\eta_{c}+\gamma_{t}+\varepsilon_{ct}$$

(4) $$Invent_{ct}=\beta_{0}+\beta_{1}DID_{ct}*Market_{ct}+\beta_{2}Control_{ct}+\eta_{c}+\gamma_{t}+\varepsilon_{ct}$$

In Equation (2), $HC_{ct}$ is the level of human capital in city c, measured by the number of employed people with education level above specialist in city c. This is to explore whether there is any difference in the effect of carbon emissions trading policies in cities with different levels of human capital. In Equation (3), $IPR_{ct}$ denotes the intensity of intellectual property (IPR) protection in city c, measured by the value of technology market transactions in city c/GDP of city c, to explore whether there are differences in the effects of carbon emissions trading policies in cities with different levels of intellectual property protection. In Equation (4), $Market_{ct}$ denotes the degree of marketization of city c, expressed as the marketization index of city c. It mainly measures whether there are differences in the effects of carbon emissions trading policies in cities with different degrees of marketization.

In order to further analyze the existence of spillover effects of the policy to the neighboring areas of the pilot areas, dummy variables are set (*Near_c_*) within the sample area; assuming that the neighboring provinces of the carbon emissions trading pilot area are 1 and otherwise should be 0, the following model is constructed.

(5) $$Invent_{ct}=\alpha_{0}+\alpha_{1}Near_{c}\times post_{t}+\alpha_{2}Control_{ct}+\eta_{c}+\gamma_{t}+\varepsilon_{ct}$$

In Equation (4), if $\alpha_{1}$ is significantly positive, then there is positive spillover effect of the policy to neighboring provinces.

### 4.4. Parallel Trend Test

The assumption of parallel trends is an essential condition for the use of the differences-in-differences method, which requires that the level of green technology innovation in the treatment and control groups must maintain a common trend prior to the implementation of the carbon emissions trading policy. In order to test whether the baseline regressions in this paper satisfy the parallel trend, this paper uses event analysis to verify whether the parallel trend test holds.

**Figure 1:**
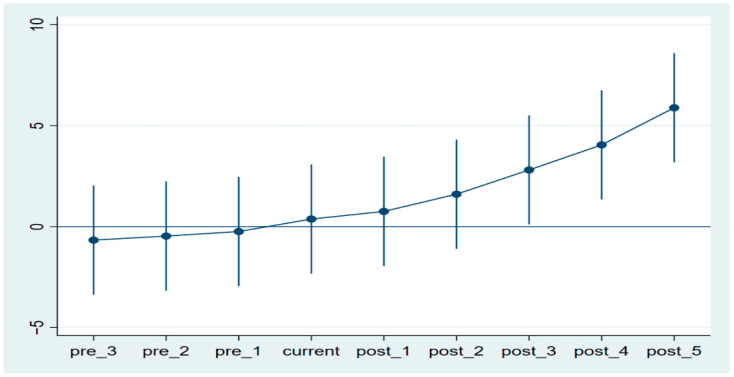
Parallel trend test chart.

Figure 1 shows that the regression results are insignificant in the years before the pilot policy, and become significant and positive two years after the pilot policy year, indicating that there is a two-year lag effect in the pilot carbon trading policy. Another reason is that it takes time to develop innovations; therefore, there is a lag effect. The regression results started to be significant two years after the implementation of the carbon emissions trading policy and the impact on regional technological innovation gradually increased.

## 5. Empirical Results

### 5.1. Baseline Regression

To test hypothesis 1, this paper used baseline regressions to primarily estimate the combined effect of carbon emissions trading policies on green technology innovation. The regression results are shown in Table 2, column (1) without the addition of control variables and column (2) with the addition of control variables. The regression results indicate that there is a significant positive effect of the carbon emissions trading system on green technology innovation at the 1% level without the addition of control variables, and also at the 1% level with the addition of control variables; therefore, with or without the addition of control variables, the carbon emissions trading system can significantly promote the level of green technology innovation.

### 5.2. Robustness Tests

**Figure 2:**
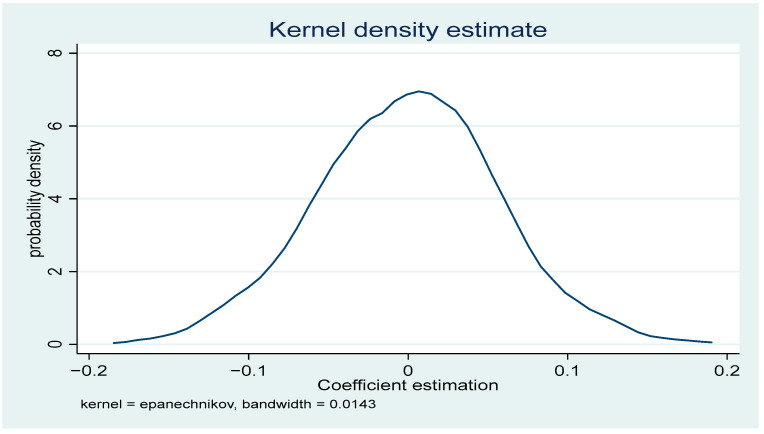
Placebo test.

In order to further test the robustness of the regression results, this paper uses two methods to conduct robustness checks. As shown in column (1) of Table 3, the coefficient of did_1 is not significant when the policy is changed in the model, indicating that the carbon emissions trading system implemented in 2013 is effective and there is no policy effect before the implementation of the carbon emissions trading policy; thus, the previous baseline regression results can be considered to have passed the robustness test. Second, considering the problem of serial autocorrelation, if the model has serial autocorrelation, it may lead to low standard errors of the DID model estimation and easily over-reject the original hypothesis. Therefore, this paper uses Block bootstrap to repeat random sampling 500 times to alleviate the problem of inconsistent standard errors of the estimated coefficients due to serial correlation. Column (2) of Table 3 presents the results obtained from the estimation using the Block bootstrap method; the results indicate that a carbon trading system can significantly increase the level of green innovation technology, and the conclusions verified in the previous paper still hold true. Placebo test refers to Ferrara [28]. In order to exclude the economic incentive effect of the carbon trading pilot from being confounded by other unobserved omitted variables, this paper performs an indirect test by randomly selecting seven samples from the full sample as the treatment group, while using (2) in Table 1 as the baseline results. To improve the identifiability of the placebo test, this random process is repeated 500 times in this paper, and the probability density distribution of the estimated coefficients is reported in Figure 2. It can be found that the randomly assigned estimates are concentrated around zero and the benchmark estimates lie outside the entire distribution, suggesting that there is no policy effect of the randomly set up carbon trading and, conversely, that the 2013 carbon trading pilot has been a significant catalyst for green technology innovation.

### 5.3. Heterogeneity Analysis

As shown in Table 4, the coefficient of Did*HC in column (1) of the regression results is positive and significant at the 1% level, indicating that in the pilot cities of carbon emissions trading, the stronger the human capital, the better the effect of the carbon emissions trading system; further, the stronger the human capital, the easier it is to create a good competitive condition for green technological innovation and to lay a certain foundation for green technological innovation, which can achieve Pareto.

The regression results in column (2) of Table 4 indicate the impact of the degree of marketization on the carbon emissions trading system, where the $DID_{ct}*Market_{ct}$ coefficient is significantly positive at the 1% level, indicating that the degree of marketization in a region has a positive impact on the carbon trading system, increasing green technology innovation. A region with a relatively high degree of marketization makes it easier to sell the carbon credits saved through innovation, and firms have more incentives to invest; thus, good conditions have been created for green technology innovation.

The regression results in column (3) of Table 4 indicate the effect of the intensity of IPR protection on the effectiveness of the carbon trading system, where the ${\mathrm{DID}}_{\mathrm{ct}}{*\mathrm{IPR}}_{\mathrm{ct}}$ coefficient is significantly positive at the 1% level but its coefficient is small, indicating that there is a positive but weak effect of the policy. It indicates that stronger intensity of IPR protection in a region will make the carbon emissions trading system increase the effect of technological innovation. The possible reason is that the stronger the IPR (intellectual property right) protection, the greater the cost of innovation patents being imitated, and the more willing companies are to invest in research to innovate, thus promoting the carbon emissions trading system to promote green technological innovation. However, the coefficient is small, indicating a positive but weak policy effect.

### 5.4. Analysis of Spatial Spillover Effects

For the spillover effect on neighboring provinces, corresponding to column (4) in Table 4, it can be seen that the coefficient of $Near_{c}*post\_t$ is insignificant, indicating that there is no spillover effect of the policy of carbon emissions trading system on green technology innovation to the collar provinces, probably because the policy of carbon emissions trading policy is not a mandatory policy now involving fewer enterprises, and is too short to cause a spillover effect.

### 5.5. Analysis of Mechanisms

As an important institutional innovation in the field of environmental regulation, the special feature of the carbon emissions trading system is that enterprises allocate carbon resources through the trading of emission rights in the carbon market; when their carbon emissions exceed the given carbon quota, they need to buy carbon emission rights in the market to make them comply with the policy. The above empirical analysis has verified that carbon trading policies significantly promote regional green technology innovation; however, how do the intermediate mechanisms of carbon trading policies affect regional green technology innovation? Does it affect regional green technology innovation by influencing R&D investment? This is the next question that will be addressed. Hypothesis 3 is proposed: carbon emissions trading policies will indirectly influence regional green technology innovation by affecting R&D investment. Drawing on the test for mediating effects, the following model is constructed [29].

(6) $$Invent_{ct}=\beta_{0}+\beta_{1}DID_{ct}+\beta_{2}Control_{ct}+\eta_{c}+\gamma_{t}+\varepsilon_{ct}$$

(7) $$Input_{ct}=\alpha_{0}+\alpha_{1}DID_{ct}+\alpha_{2}control_{ct}+\eta_{c}+\gamma_{t}+\varepsilon_{ct}$$

(8) $$Invent_{ct}=\delta_{0}+\delta_{1}\;DID_{ct}+\delta_{2}Input_{ct}+\delta_{3}Control_{ct}+\eta_{c}+\gamma_{t}+\varepsilon_{ct}$$

where *Input* is the mediating variable in this model, denoting research inputs. Column (1) is the baseline regression and $\beta_{1}$, the coefficient of the regression, is significantly greater than 0, indicating that carbon emissions trading policies can positively promote regional green technology innovation; column (2) verifies the effect of carbon emissions policies on the level of research investment, and the coefficient of the regression is significantly positive. $\alpha_{1}$, the coefficient of (2), verifies the effect of carbon emissions policy on the level of research investment, which is significantly positive, indicating that carbon emissions trading policy can significantly increase the level of research investment of enterprises; column (3) verifies the effect of research investment level on regional green technology innovation, which is significantly greater than 0. $\delta_{1}$, the coefficient of (3), is significantly greater than zero, indicating that the higher the level of investment in research, the more it can promote the level of green technology innovation in the region.$\delta_{1}<\beta_{1}$, indicating that there is a partial intermediary effect.

In Table 5, the statistical results in columns (1), (2), and (3) show that the mediating effect of research investment is in line with the expected hypothesis. Carbon emissions trading policies can indirectly affect the level of regional green technology innovation through influencing the level of research investment.

## 6. Discussion

Based on the above analysis, the policy implications in this paper are as follows:

First, develop a multi-level carbon trading market and increase the activity of the carbon trading market. A single type of carbon trading market can lead to the problem of insufficient activity of the carbon trading market. Therefore, social forces should be actively encouraged to join the field of emission reduction, form multi-level and diversified greenhouse gas emission reduction synergies, and enhance market vitality.

Second, improve the legislation on carbon emissions trading so that the development of the carbon trading market has a law to follow. China’s carbon emissions trading market pilot began in 2013; after 10 years of development, the policy documents on the carbon trading market are outlines, plans, guidance, notices, etc., and have not yet been strengthened by legislation, so we should accelerate the formulation of the Energy Law, Climate Change Law, and other relevant laws to effectively safeguard and protect the legitimate rights and interests of various market entities.

Third, strengthen the protection of regional intellectual property rights and reduce the cost of public rights protection. The stronger the intensity of regional intellectual property protection, the lower the probability of innovation patents being imitated, and the more willing enterprises will be to invest in R&D funds for innovation. Relevant departments should further raise the standard of infringement compensation, and comprehensively use methods such as review authorization, administrative law enforcement, judicial protection, and arbitration mediation, to form a joint force for intellectual property protection and to better combat and deter all kinds of infringement. The government should build a number of intellectual property protection centers to provide the public with more convenient, efficient, and low-cost channels for rights protection.

Fourth, cultivate high-quality human capital and enhance the ability of green technology innovation. High-quality human capital is the foundation for technological innovation, and China should continue to increase the proportion of education expenditure and R&D expenditure in GDP; give full play to the synergy between the government, enterprises, schools, and markets; and accelerate China’s breakthroughs in green technologies such as energy conservation and consumption reduction, carbon capture, and carbon storage.

Fifth, innovate carbon finance tools and expand investment and financing channels for green projects. The active participation of finance is crucial to the construction of China’s carbon trading market. Although China’s financial institutions have successively launched carbon financial products such as carbon funds, carbon bonds, and carbon options, due to the imperfection of the current carbon financial market system, the carbon trading market is illiquid enough to meet the needs of trading entities. To this end, it is suggested to stimulate the development potential of the carbon trading market through financial support and policy guidance, promote the financialization of the carbon trading market, and improve the liquidity and market activity of the carbon trading market by amplifying the financial attributes of carbon emission rights. In addition, carbon financial instruments are continuously being innovated to enhance liquidity, and price control mechanisms are being established to keep carbon prices stable.

## 7. Conclusions

This paper studies the impact of the carbon emissions trading system on regional green technology innovation by using the data of provincial and regional green technology innovation and the double difference method; further, it analyzes whether there are spillover effects of carbon emissions trading policies on neighboring areas of the pilot areas and whether there are differences in the effect of carbon emissions trading policies in promoting green technology innovation in different regions.

The conclusions of this paper show the following: (1) The carbon emissions trading system can significantly promote the level of regional green technology innovation, and there is a lagging effect in the policy, which is still valid after the robustness test, confirming the existence of the Porter hypothesis. (2) There is no regional spillover effect of the ETS on neighboring regions. (3) The carbon emissions trading system works better in regions with stronger human capital, which can significantly enhance the effect of carbon emissions trading policies on regional green technology innovation. (4) The stronger the protection of intellectual property rights, the greater the impact of the carbon emissions trading system on regional green technology innovation. (5) In regions with a relatively high degree of marketization, it is easier to sell carbon emission allowances saved through innovation, and the more incentive mechanisms enterprises have to invest, which creates good conditions for green technology innovation.

## Figures and Tables

**Table 1:** Descriptive statistics.

Variables	Mean	SD	Min	Median	Max
*DID*	0.65	0.25	0	0	1
*Open*	0.31	0.36	0.01	0.14	1.68
*Scale*	3838.01	10,488.99	0.57	2.79	65,303.97
*GDP*	17,676.14	17,494.03	243.09	12,170.80	107,986.90
*STR*	0.46	0.09	0.30	0.45	0.84
*HC*	15.18	10.02	0.25	13.36	62.19
*Tec*	79,752.91	115,294	22	42,632.5	700,017
*Invent*	2527.86	4659.67	0.00	776.00	31,391.00
*Market*	6.26	2.09	−1.14	6.21	10.92
*Input*	1,010,000	606,040.07	23,617.49	86,1523.00	3,240,000
*IPR*	1,140,000	2,300,000	29.44	343,577.59	16,100,000

**Table 2:** Baseline regression results.

	(1)	(2)
	*Invent*	*Invent*
*DID*	5.076 ***	1.342 ***
	(7.55)	(3.18)
*Control*	No	Yes
*Year*	Yes	Yes
*Area*	Yes	Yes
*_cons*	2.200 ***	1.556
	(16.61)	(0.98)
*Observations*	465	445
*R* *-Squared*	0.697	0.902

Note: *** indicate that the regression coefficient is significant at the levels of 1%, with t-stats in parentheses.

**Table 3:** Robustness tests: timing of policy change onset and mitigation of serial correlation issues.

	(1) Policy Point Is 2012	(2) Bootstrap
	*Invent*	*Invent*
*did_1*	0.560	
	(1.42)	
*DID*		1.342 **
		(2.00)
*control*	Yes	Yes
*Year* *Area*	Yes Yes	Yes Yes
*_cons*	2.012	1.556
	(1.27)	(0.78)
*No. Observations*	445	445
*R* *-Squared*	0.900	0.902

Note: ** indicate that the regression coefficient is significant at the levels of 5%, with t-stats in parentheses.

**Table 4:** Heterogeneity analysis and spatial spillover effects.

	(1)	(2)	(3)	(4)
	*Invent*	*Invent*	*Invent*	*Invent*
$DID_{ct}*HC_{ct}$	0.072 ***			
	(5.97)			
$DID_{ct}*Market_{ct}$		0.128 ***		
		(2.80)		
$DID_{ct}*IPR_{ct}$			0.005 ***	
			(9.33)	
$Near_{c}*post\_t$				0.495
				(1.37)
control	Yes	Yes	Yes	Yes
Year	Yes	Yes	Yes	Yes
Area	Yes	Yes	Yes	Yes
_cons	0.335	0.690	−1.852	2.328
	(0.21)	(0.42)	(−1.13)	(1.47)
*No*. *Observations*	414	401	430	445
*R* *-Squared*	0.917	0.911	0.920	0.900

Note: *** indicate that the regression coefficient is significant at the levels of 1%, with t-stats in parentheses.

**Table 5:** Analysis of mechanisms.

	(1)	(2)	(3)
	*Invent*	*Input*	*Invent*
*DID*	3.626 ***	6.43 ***	2.837 ***
	(7.09)	(3.42)	(6.08)
*Input*			0.001 ***
			(8.69)
*control*	Yes	Yes	Yes
*Year*	Yes	Yes	Yes
*Area*	Yes	Yes	Yes
*_cons*	−1.271	−22.37 ***	1.476
	(−0.54)	(−2.60)	(0.70)
*No*. *Observations*	341	341	341
*R* *-Squared*	0.891	0.975	0.913

Note: *** indicate that the regression coefficient is significant at the levels of 1%, with t-stats in parentheses.

## Data Availability

Not applicable.
